# Describing healthcare providers’ perceptions of relational practice with families in the emergency department: A qualitative study

**DOI:** 10.4102/curationis.v43i1.2155

**Published:** 2020-11-02

**Authors:** Waheedha Emmamally, Christen Erlingsson, Petra Brysiewicz

**Affiliations:** 1 Discipline of Nursing, College of health Sciences, University of KwaZulu-Natal, Durban, South Africa; 2 Department of Health and Caring Sciences, Faculty of Health and Life Sciences, Linnaeus University, Kalmar, Sweden

**Keywords:** emergency departments, families, healthcare professionals, families, perceptions, relational practice

## Abstract

**Background:**

Emergency departments are regarded as stressful working environments, associated with staff shortages, increased patient numbers and long waiting times. Increased organisational demands for performance can compromise genuine interactions between families and healthcare providers working in emergency departments. A relational practice approach in caring for families can enhance the capability of healthcare providers to simultaneously overcome these difficulties and provide emergency healthcare of high quality.

**Objectives:**

The purpose of the study was to describe healthcare providers’ perceptions of relational practice with families in three emergency departments in KwaZulu-Natal, South Africa.

**Method:**

Using a qualitative descriptive approach data were collected through semi-structured interviews with healthcare providers working in emergency departments. The data were analysed and categorised using qualitative content analysis.

**Results:**

Four categories emerged from data analysis: (1) families and healthcare providers connecting; (2) recognising the uniqueness of families; (3) caring interactions; and (4) taking charge when necessary.

**Conclusion:**

The study elicited that healthcare providers working in emergency departments perceived that despite high patient volumes and resource constraints, collaborative relationships with families were important. However, these collaborative relationships cannot be willed into practice, instead training workshops are needed to develop relational skills of healthcare providers which can facilitate family and healthcare professional collaboration.

## Introduction

For healthcare providers (HCPs), the complexity of the emergency department (ED) makes it a challenging context in which to collaborate and engage with families accompanying a patient (Rodgers 2015). Some of the factors that impeded effective family and healthcare professional collaborations are organisational expectations for efficient patient flow through the ED, the episodic nature of the emergency visit, and perceived urgency of treatment as understood by families and HCPs alike (Hsiao et al. 2017; Suroso et al. 2015). Other difficulties in collaborating with families include linguistic and cultural diversity and lack of a pre-established relationship between the HCPs and families (Dudley et al. 2015).

In developing countries, the provision of quality emergency care to patients and families is further compounded by the lack of resources, especially limited numbers of experienced HCPs and limited space in which to treat patients (Atakro et al. 2018). Under these circumstances, HCPs working in the ED struggle with overcrowding and prioritising emergencies, and have little time to collaborate and connect with families (Botes & Langley 2016). However, research findings link positive patient and family experiences of care to improved clinical outcomes (Chatterjee, Tsai & Jha 2015; Indovina et al. 2016), making collaborative engagements between families and HCPs a necessity rather than an option of care (Goodridge, Isinger & Rotter 2018; Letvak & Rhew 2015; Morphet et al. 2015; Williams, Nolan & Keady 2009). Thus, HCPs working in the ED must reach beyond workload pressures to practices that promote collaborating with families. Relational practice with its emphasis on growth through connecting with others is viewed as the conduit to achieving this (Frampton et al. 2017; Jordan 2010).

Relational practice in healthcare is about understanding patients and families in their unique socio-economic, cultural and political context, and appreciating how this context affects their relationships and experiences, which includes the ED experience (Lenz 2016). Fyers and Greenwood (2016) assert that relational practice is not a standardised way of responding, but rather to respond or not to respond depends on the individual and the situation. Relational practice focuses on creating authentic connections with families, where the HCP is fully present in his or her engagement with the patient and the family and is able to respond in ways that are culturally appropriate to the family (Doane & Varcoe 2007; Zou 2016). Other key elements in relational practice with families are the desire to connect with families, mutual respect and empathy in the family–HCP interactions and for HCPs to identify and confront subjectivity in their practices (Hartrick Doane & Varcoe 2015; Zou 2016). Bearing in mind their instrumental role in collaborating with families in the ED, HCPs perceptions and experiences of relational practice represent a vital dimension in promoting relational practice in the ED (Braganza 2017).

## Purpose of the study

The purpose of this study was to describe HCPs’ perceptions of relational practice with families in three EDs in KwaZulu-Natal, South Africa.

## Methods

A qualitative descriptive research design was chosen, as it facilitated an in-depth exploration of the multiple realities of the HCPs on relational practice.

### Study setting and respondents

The study was conducted in two EDs of state-funded hospitals and one ED within a private hospital in KwaZulu-Natal (KZN). South Africa has a two-tier healthcare system, comprising state-funded hospitals that provide healthcare services to individuals who do not have health insurance, and private hospitals that provide healthcare to those who can afford the services or have private health insurance (Young 2016). The state-funded hospitals provide healthcare to 80% of the population who reside in rural, urban and unplanned, temporary housing settlements, surrounding the hospitals (Dell & Kahn 2017). According to Krugg and Alarcos (2017), the two-tiered healthcare system is characterised by mal-distribution of resources where private hospitals are generally better equipped in terms of resources, staffing and infrastructure. By way of comparison a medical doctor (MD) in a private hospital provides services to approximately 500 people in comparison to approximately 11 000 people in the public sector. State-funded hospitals are characterised by low staffing levels, unqualified HCPs and inadequate resuscitation and diagnostic facilities (Hardcastle et al. 2016; Jerome et al. 2017). These disparities do not only reflect resource shortages but may have an adverse impact on HCPs’ attitudes and perceptions regarding their practice leading to poor quality care (Maseko & Harris 2018).

Healthcare providers were purposively sampled based on them being involved in direct forms of patient care. Inclusion criteria for participation were the following: MDs (with or without a specialisation in emergency medicine) and nurses registered with the South African Nursing Council as an enrolled nurse (EN) with a 2-year certificate or as a professional nurse (PN) with a diploma and/or degree in nursing (with or without a specialisation in emergency nursing) made the sample. During data collection in the quantitative component of the larger mixed methods study, participants were asked to indicate whether they would be willing to continue their involvement in the study by being interviewed at a later stage. Those agreeing to participate further provided their details on the questionnaire and were contacted by the first author. The study recruited nine HCPs.

### Data collection

The first author (W.E.) conducted one semi-structured interview with each participant during August and September 2017. The question guiding the interview was: ‘Describe your perception of relational practice with families in the ED’. Interviews took place during the participants’ off-duty times, on a day and time requested by them, in a room within each setting where the interviewer and participant would not be disturbed. Interviews lasted approximately 30–40 min and were audio-recorded with permission from the participants. After discussion with the other authors (C.E., P.B.), a decision was made to stop data collection after the nine interviews as no new findings were emerging and participants were describing similar perceptions of relational practice.

### Analysis

The interviews were transcribed verbatim and analysed using content analysis guidelines as described by Erlingsson and Brysiewicz (2017) and Graneheim and Lundman (2004). Three independent coders (W.E., C.E., P.B.) read each transcript several times and primary codes were derived from the condensed meaning units. The three coders met to discuss the emergent codes and any discrepancies in the codes were resolved. Codes with similar content and meaning were grouped to form categories.

### Trustworthiness

Measures for credibility, transferability, conformability and dependability were incorporated in the study to achieve trustworthiness (Lincoln & Guba 1985; Shenton 2004). To ensure credibility all interviews were audio-recorded and member checking with HCPs was achieved whereby, after transcribing the interviews, the researchers went back to the HCPs to confirm that the emerging findings were a representation of their reality. Credibility was also enhanced through independent analysis where the three authors independently analysed the data (Noble & Smith 2015). Although the study, being qualitative in approach, was not aiming for generalisability of findings, detailed descriptions of the participants, research settings, time frame and data collection methods were presented to allow readers to make their own decisions regarding transferability of study. To achieve conformability, the researcher maintained an audit trail during the interview process. Dependability was achieved by the researcher providing a comprehensive methodological description to allow for the replication of the study.

### Ethical consideration

The study received ethical approval from Humanities and Social Sciences Research Ethics Committee of the University of KwaZulu-Natal (HSS/1731/015D) and the ethics committee of the private hospital (251015).

Participants gave written informed consents prior to being interviewed, and were briefed about the study and their right to withdraw from the study without risking reprisal. The transcribed interviews were stored in electronic folders that were password protected on a secure university server.

## Results

The final sample consisted of four PNs, two ENs and three MDs. Of the four PNs, three worked in state-funded hospitals (PN1-3, state) and one in the private hospital (PN1, private). All four of the PNs had a specialist qualification in emergency care nursing. Both the ENs worked in state-funded hospitals (EN1 and 2, state). Of the MDs, two worked in state-funded hospitals (MD1 & 2, state) and one in the private hospital (MD3, private). Two of the MDs had a specialisation in emergency care. Two of the MDs and two PNs were male and both ENs were female. The participants described their perceptions on relational practice in the context of their interactions with families in the ED. Four categories emerged from the content analysis: families and HCPs connecting; recognising the uniqueness of families; caring interactions; and taking charge when necessary.

### Families and healthcare providers connecting

Participants likened relational practice to building a bridge that connects HCPs with families, in which either party could easily reach out to the other. This was explained as:

‘Constructing a bridge together, where the stronger the bridge the easier to cross over to each other.’ (PN 4, state, female)

The ED nurses were seen as the relationship builders with families. Healthcare providers stated that families were uncomfortable when communicating with doctors and the nurse often interceded on the family’s behalf:

‘Families are generally scared … to talk to doctors, so nurses are the bridge to facilitate these interactions.’ (MD 3, private, male)

Participants expressed that building effective relationships between families and HCPs meant that families and HCPs need to work together to provide quality care for the patient.

‘We work with families and not against them for a common purpose.’ (MD 1, state, male)

### Recognising the uniqueness of families

Healthcare providers stated that relational practice was about understanding that each family was uniquely different by virtue of their cultural beliefs and practices, language and socio-economic circumstances. The participants added that each family responded differently during the ED admission, as influenced by their beliefs and circumstances, and the care offered by HCPs needed to be aligned to the family’s responses and cultural preferences.

‘Interact with them [*families*] so as to understand where they are coming from, support them in what they need and not what we assume that they need. Know that each family requires a different type of interaction with us health workers, and each family will react differently.’ (PN 3, state, male)

Participants spoke of respecting families’ values and cultural beliefs, especially when communicating and offering treatment options to families. One participant elaborated:

‘We were taught that a good communication skill is to look directly at a person when talking to them, but in some cultures this is a sign of disrespect. So I think relational practice is looking at what is culturally acceptable to the family.’ (PN 1, state, female)

A HCP expressed that it was important to respect a family’s decisions regarding their extent of being involved in their loved one’s care. The participant stated:

‘When a new trend comes about in caring for patients then we go to extremes to follow it…We must understand that not all families want to make decisions for the patient. They believe that to do so is intrusive to a patient. Again their involvement is dictated by their culture, and we have to respect their wishes.’ (MD 3, state, male)

### Caring interactions

Participants explained that factors of overcrowding, insufficient time and lack of resources often made it difficult for them to be able to care for families in the ED. However, the majority of the HCPs indicated that from their experience in the ED, they have come to realise that families did not expect a great deal from the HCP and appreciated simple gestures of care. These gestures did not in fact require many resources nor a great deal of time. A participant described this in the following statement:

‘I have water here, tissues, and we have some frames [*pictures*] about hope and so on. This helps to calm them. In my time in the ED I realise that simple things mean the most to people who are suffering.’ (MD 2, state, female)

Participants indicated that communicating with families was a vital part of relational practice. Healthcare providers agreed that the different languages of families had in the past led to miscommunication of important information regarding the patient. They elaborated that this barrier to communication was solved by, ‘*hospital staff speaking different languages have volunteered themselves as interpreters so even a language like Tswana or Venda is* understood’ (PN 2, state, male). Participants added that providing clear, simple explanations to families and being mindful of the tone of voice were important aspects in communicating with families. A HCP stated:

‘When we communicate, we have to be attentive as this shows that we are willing listeners. Communication must be simple and given in a calming tone of voice. We want to clear their doubts not add to it.’ (PN 4, private, female)

Healthcare providers indicated that part of relational practice was to reflect on their interactions with families and ask for assistance from colleagues who were seen to have greater skills in collaborating with families. As one participant revealed:

‘It takes a staff member who has that special way to communicate to relate to families. And what I have realized (laughs) is that we all do not have these skills, so be aware of your limitations and call on the staff who do have these skills.’ (EN 1, state, female)

Participants spoke of a need for training workshops to develop a repertoire of skills to interact successfully with families. A participant expressed:

‘We are coping with so many situations and so many emotions. Working in an emergency department gives you experience to improve your clinical skills. There must be training programmes in place to equip us with skills of understanding and of empathy and more importantly how to use these skills.’ (PN 3, state, male)

### Taking charge when necessary

Two participants expressed that relational practice with families included HCPs taking charge of the ED admission, as families were unable to cope on their own and were dependant on HCPs. A participant stated:

‘Families are weak and overwrought. They rely on us [HCPs]. We are always accused of taking charge, but in these situations, we know what is best.’ (EN 2, state, female)

A participant indicated that HCPs had to make health decisions for families as families did not always have the required knowledge to make their own decisions.

‘We meet families from different educational backgrounds, and they are not equipped to make decisions about the patient. We need to step in and make decisions that are in their interest.’ (MD1/state)

## Discussion of the results

The participants in the present study expressed that relational practice was about HCPs and families working together to improve the care of the patient. They linked relational practice to partnerships between families and HCPs. Huang (2014) and Tapp (2000) have similar views of relational practice as partnership between families and HCPs, characterised by open dialogue, mutual caring and non-prescriptive HCP behaviour. Baretto et al. (2018) and Frampton et al. (2017) revealed contradictory findings of families being excluded from participating in the patient’s care and families being ignored by HCPs. With some research findings highlighting family exclusion in EDs (Dias 2017), it is important that HCPs in this study did acknowledge that collaborating with families is fundamental to positive family outcomes in the ED.

Participants spoke of ED nurses’ facilitating family–doctor interactions. The notion of nurses as the family liaison is a frequent finding in the literature, with nurses seen as drivers in relating and connecting with families in the wider health sector (Misto 2014; Olley et al. 2016). Whilst it is important to acknowledge the role played by ED nurses in supporting families cope with the complexities of the ED experience, the commitment of a multidisciplinary role to relational practice is necessary in improving healthcare outcomes.

Healthcare providers reported that acknowledging a family’s uniqueness was an important aspect of relational practice with families. Zou (2016) agreed that families often respond differently to both illness and the ED admission and there is therefore a need for HCPs to discuss and negotiate aspects of care with each family according to their unique needs. The focus on family’s unique beliefs, experiences and preferences when providing care is a finding that must be incorporated in all aspects of care to improve collaboration with families (Shields 2015).

Healthcare providers in the current study took the view that relational practice means respecting families’ beliefs and values, not only on illness and health but their values on relationships with people. Although many studies reveal that HCPs recognise that respect for families is fundamental to all positive interactions with them (De Beer & Brysiewicz 2017; Emmamally & Brysiewicz 2018; Segaric & Hall 2015), it is acknowledged that respecting families who have different values and cultural beliefs can be difficult to achieve (Upasen 2017).

Through relational practice, HCPs must create healthcare structures that respect the cultural beliefs of families and meet the cultural preferences of families in culturally safe ways (Fyers & Greenwood 2016).

It seems to be important that HCPs commit to approaches of interested inquiry and attentive listening, so not to lose sight of the cultural diversity of families with whom they interact.

Participants believed that families should be the guideposts in defining their involvement in the care of their loved one. Tse, Hung and Pang (2016) reported similar findings of emergency nurses encouraging families to express their preferred level of involvement in treatment options. Healthcare provider convictions, however, well intended that families should guide the care process and share decision-making needs to be considered realistically in the context of the ED environment – one that is full with challenges in which the pre-emptive goal is stabilisation of the patient. These pressures aside, HCPs are nonetheless called to create opportunities for families to ask questions and participate in decision-making (Grudzen et al. 2016; Hess et al. 2015). Suggestions by study participants on respect for the unique needs of each family and being responsive to a family’s wishes for involvement indicate directions to follow in engaging and collaborating with culturally, politically and socio-economically diverse families.

Though the ED was cited as overcrowded, with severe staff shortages that limited the time for collaborating with families, the HCPs in the present study indicated that families appreciated simple gestures of care which could be done in the minimal of time, with the least of resources. Contrary findings were found in a study in Ghana, where registered nurses said that the overcrowding and resource restraints in the ED impacted their ability to give quality care to families (Atakro et al. 2018). Cypress (2014) and Sinclair et al. (2016) in different studies concluded that patients and families appreciated any gestures of kindness from HCPs aside from routine care. Resource limitations acknowledged that it is nonetheless important that HCPs understand that the briefest of moments can be utilised to genuinely connect.

In keeping with research findings of therapeutic communication and family discussions as essential aspects of relational practice with families (Loghmani, Borhani & Abbaszadeh 2014), participants in the current study expressed a wish for training and support in developing skills needed to communicate and collaborate with families. The HCPs’ request for skills training workshops to promote their working with families is a significant finding as there is a general recognition that training programmes can be invaluable in developing relational skills and strategies to collaborate with families (Coats et al. 2018; Coyne et al. 2011).

Self-awareness is seen as an important element of relational practice because it enables health practitioners to develop a deeper understanding of themselves and situations (Koshy et al. 2017). Similarly, participants in the current study believed that reflecting on their abilities to interact with families was important. Through reflection they were able to seek assistance from colleagues who were better equipped in interacting with families. These findings confirm the importance of reflective practice in helping HCPs gain insight into their behaviours and to align these behaviours to the elements of relational practice.

Some participants in the study indicated that relational practice in the ED was about HCPs taking charge in helping families to cope. Healthcare providers stated that families in the ED were either too overwrought to make decisions or, because of their educational background, ill-equipped to make decisions. These findings are contradictory to a relational practice approach of true collaboration with families, where HCPs must concede that the emphasis is not on acting for families, but on working with families and empowering them to be vocal in their care (Frampton et al. 2017).

## Recommendations

Based on the outcomes of this study, the following recommendations are advocated for clinical practice and policy, nursing education and nursing research, respectively:

It is recommended that academic and clinical practitioners collaborate to identify the learning needs of clinical staff with regard to their relational practice with families. Workshops and in-service programmes must be designed to focus on the training needs of HCPs with regard to developing their relational practice with families. There is a recommendation that clinical policies regarding families be revisited to make sure that these policies can be implemented within the constraints of the ED setting.

Important suggestions for nursing education include aligning nursing curricula for both undergraduate and postgraduate programmes to focus on relational skills and reflective inquiry in nurses. Such activities may encourage nurses to reflect on elements of relational practice when interacting with families. Capacity-building initiatives for academics should be in place to ensure that they have the necessary relational skills training to facilitate students.

The study findings indicated that the HCPs believed that their extreme workload impedes their relational practice with families in the ED, hence the authors recommend a qualitative inquiry on the impact of this workload – specifically how the ED workload impacts HCPs’ relational practices with families.

## Strengths and limitations of the study

Previous studies in the ED focused on improving technical competencies to improve quality of care. This study provided a rare insight into the perceptions of HCPs on connecting and engaging with families. In doing so, it may catapult more research into relationships in the ED as a means to improving quality of care rendered to patients and their families. A noted limitation of the study is that the participants interviewed were nurses and MDs working in the ED. The results may therefore not be representative of perceptions of other categories of staff in the ED, namely, prehospital staff, clerks and porters. Bearing in mind the subjective nature of qualitative research, results of the study should be applied with caution to populations not included in the study.

## Conclusion

Several important aspects emerged from HCPs’ perceptions of relational practice when interacting with families in ED. First, though HCPs alluded to the challenges of overcrowding and resource shortages in the ED impacting on the time available to engage with families, they conceded that families appreciated simple gestures that were not time-intensive. Second, HCPs needed to create meaningful connections with families that are founded on respect and an understanding of how contextual factors can influence a family’s response to the ED admission. Third, in assisting families with treatment decisions there must be due recognition of their desired level offer involvement in the care process. The study also provides an understanding of HCPs’ relational practice with families from diverse backgrounds and socio-economic settings.
